# Processed Eggshell Membrane Powder Is a Promising Biomaterial for Use in Tissue Engineering

**DOI:** 10.3390/ijms21218130

**Published:** 2020-10-30

**Authors:** Sissel B. Rønning, Ragnhild S. Berg, Vibeke Høst, Eva Veiseth-Kent, Christian R. Wilhelmsen, Eirik Haugen, Henri-Pierre Suso, Paul Barham, Ralf Schmidt, Mona E. Pedersen

**Affiliations:** 1 Nofima AS, Pb 210, NO-1431 Ås, Norway; sissel.beate.ronning@nofima.no (S.B.R.); ragnhild.berg@nofima.no (R.S.B.); vibeke.host@nofima.no (V.H.); eva.veiseth-kent@nofima.no (E.V.-K.); christian.ringnes.wilhelmsen@gmail.com (C.R.W.); eirik_rh92@hotmail.com (E.H.); 2 Biovotec AS, Postbox 1001 Hoff, 0218 Oslo, Norway; henri@biovotec.com (H.-P.S.); paul@biovotec.com (P.B.); ralf.schmidt@biovotec.com (R.S.)

**Keywords:** processed eggshell membrane powder, scaffold, cell migration, human dermal fibroblasts, myofibroblast, bovine muscle cells, extracellular matrix

## Abstract

The purpose of this study was to investigate the tissue regenerating and biomechanical properties of processed eggshell membrane powder (PEP) for use in 3D-scaffolds. PEP is a low-cost, natural biomaterial with beneficial bioactive properties. Most importantly, this material is available as a by-product of the chicken egg processing (breaking) industry on a large scale, and it could have potential as a low-cost ingredient for therapeutic scaffolds. Scaffolds consisting of collagen alone and collagen combined with PEP were produced and analyzed for their mechanical properties and the growth of primary fibroblasts and skeletal muscle cells. Mechanical testing revealed that a PEP/collagen-based scaffold increased the mechanical hardness of the scaffold compared with a pure collagen scaffold. Scanning electron microscopy (SEM) demonstrated an interconnected porous structure for both scaffolds, and that the PEP was evenly distributed in dense clusters within the scaffold. Fibroblast and skeletal muscle cells attached, were viable and able to proliferate for 1 and 2 weeks in both scaffolds. The cell types retained their phenotypic properties expressing phenotype markers of fibroblasts (TE7, alpha-smooth muscle actin) and skeletal muscle (CD56) visualized by immunostaining. mRNA expression of the skeletal muscle markers myoD, myogenin, and fibroblasts marker (SMA) together with extracellular matrix components supported viable phenotypes and matrix-producing cells in both types of scaffolds. In conclusion, PEP is a promising low-cost, natural biomaterial for use in combination with collagen as a scaffold for 3D-tissue engineering to improve the mechanical properties and promote cellular adhesion and growth of regenerating cells.

## 1. Introduction

Tissue regeneration and healing is an intricate process where the skin, skeletal muscle or other body tissues repair themselves after injury [1,2]. The extracellular matrix (ECM), a complex network of fibrous proteins, carbohydrates and glycoproteins surrounding the cells, is the main regulator in tissue regeneration. It works as the structural support and scaffold for cell growth and differentiation, the supplier and regulator of essential growth factors and cytokines, and cell enzyme activities (for review [3]). Tissue engineering is a promising therapeutic treatment to repair and replace destroyed tissue, where biodegradable scaffolds mimick the ECM support the regeneration process [4]. The scaffold behaves as a viable support for cells to adhere, migrate, proliferate and differentiate to fulfil their specific roles in different biological processes, and to form new tissues. An ideal scaffold would have the characteristics of excellent biocompatibility, a suitable microstructure, controllable biodegradability, and appropriate mechanical properties. Several different materials have been proposed, as a possible replacement for the ECM, with the majority being either biological or synthetic polymers such as a collagen or polyester [5]. Biological polymers are biocompatible and often bioactive, however their use has often been difficult due to the lack of control of enzymatic degradation and poor structural performance, while the usage of synthetic polymers has proven to lack biocompatibility and some materials are not biodegradable and remain in the tissue, causing an inflammatory response. Biological polymers often have low resistance to mechanical stress; hence these biomaterials are often coupled with other materials to improve the mechanical properties. One promising material in this respect is using eggshell membrane.

Eggshell membrane (ESM) is available as a by-product of the chicken egg processing (breaking) industry on a large scale, and it could have the potential as a low-cost ingredient for therapeutic scaffolds. This thin membrane inside the eggshell, serves both protective and supportive roles during eggshell mineralization [6]. We have previously demonstrated that industrially processed eggshell membrane powder (PEP) is biocompatible and has wound healing and regeneration properties in vitro and in vivo [7,8]. The structure of PEP revealed a fiber-like meshwork with a supportive possibility for cell growth. Different cell types such as fibroblasts, epithelial, and endothelial cells were stimulated for growth and differentiation by PEP. We also demonstrated that PEP has immune-modulating properties in vitro, by increasing anti-inflammatory cytokine production IL-10 and increased expression of heme-detoxification enzyme heme oxygenase-1 (HO-1), a highly expressed protein in the anti-inflammatory M2 macrophages. PEP is an equivalent to ECM, and its composition and highly structural features have been extensively characterized [9]; the meshwork of fibers is approximately 90% proteinaceous [10], and also contains complex carbohydrates such as glycosaminoglycans (GAGs) [11] and N-glycans [12]. Structural proteins such as cysteine-rich eggshell membrane proteins (CREMPS) and collagens (particularly type X) are abundant constituents, together with glycoproteins, Ca-regulatory proteins and enzymes with a disintegrin-like and metalloproteinase domain with thrombospondin type 1 motif (ADAMs) [9,13,14,15,16]. These are all constituents that are relevant from a tissue regeneration perspective [9]. 

Although collagen is widely used as biomaterial in 3D-scaffolds, improving mechanical properties is necessary due to low resistance to mechanical stress. The primary goal of the current study was therefore to investigate if PEP could be mixed with collagen in a 3D scaffold, and whether this complex bioactive material could improve the mechanical properties of the scaffolds and still retain the regeneration potential of the cells compared to pure collagen 3D-scaffolds. The structure and mechanical properties of scaffolds consisting of pure collagen or PEP as the major constituent was compared, and primary fibroblasts and skeletal muscle satellite cells, regenerators of tissues, were investigated in the study. 

## 2. Results

### 2.1. Moisture Absorption and Texture Properties of Collagen Cryo-Scaffold is Improved with PEP

**Figure 1:**
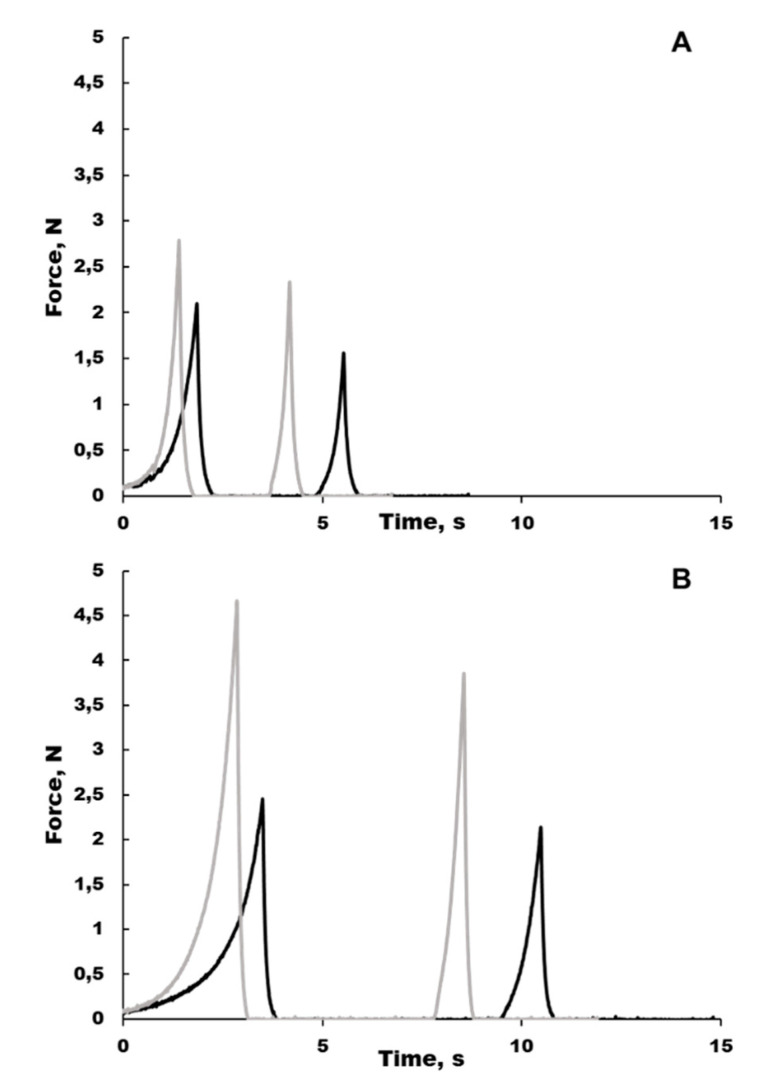
Texture profile curves from two representative collagen scaffolds without (**A**) and with (**B**) PEP following a two-cycle compression to 60% of the original scaffold height. The force (N) necessary to maintain a constant speed of the probe was recorded continuously during compression, resulting in a texture profile curve. Black curves—1 h post-swelling, grey curves—4 h post-swelling.

Inclusion of PEP in the scaffold significantly increased the absorption of phosphate-buffered saline (PBS) by approximately 50%, while the water-holding capacity (i.e., the ability to retain PBS during compression) was higher for the collagen than the PEP scaffolds and was higher at 4 h compared to 1 h post-swelling (Table 1). The height reduction caused by compression during texture analysis did not differ between the two scaffold types. The texture profile analysis (Figure 1) revealed PEP to increase the mechanical strength (i.e., hardness) of the scaffold for the 4 h post-swelling measurement (Table 1), while no differences were seen for the 1 h post-swelling measurement.

### 2.2. Scaffold with and without PEP Differ in Structure Properties

**Figure 2:**
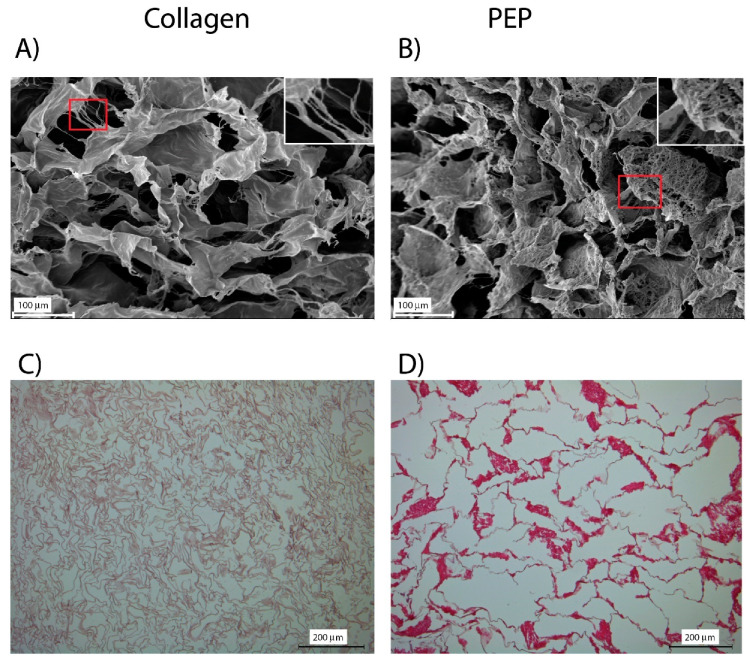
Characterization of scaffolds by SEM-analysis (**A**,**B**) and light microscopy with hematoxylin and eosin (HE)-staining (**C**,**D**). Scaffold consisting of collagen (**A**) visualized a porous structure with fibrils interconnected (larger magnification) between sheet-like structures. A similar porous structure was also obtained in scaffold-containing PEP (**B**), but with aggregates of PEP-structures (larger magnification) within. The fibril-like structures of PEP in a criss-cross pattern was more closely packed. HE-staining demonstrated an organized collagen pattern (**C**,**D**), and an even distribution of PEP-clusters of more a pinkish color (**D**).

Characterization of the scaffolds with scanning electron microscopy (SEM) demonstrated a porous structure for both scaffolds (Figure 2). Thin thread-like collagen fibrils were observed in both types of scaffolds. SEM analysis further revealed PEP as dense clusters of thread-like fibrils tightly packed. HE-staining and light microscopy analysis revealed that these PEP clusters were evenly distributed within the scaffold. More pinkish staining was also observed of PEP-particles compared to the collagen-structured material, indicating a difference in ionic-properties. 

### 2.3. Fibroblasts and Skeletal Muscle Cells Retain their Phenotypes in Both Types of Scaffold 

**Figure 3:**
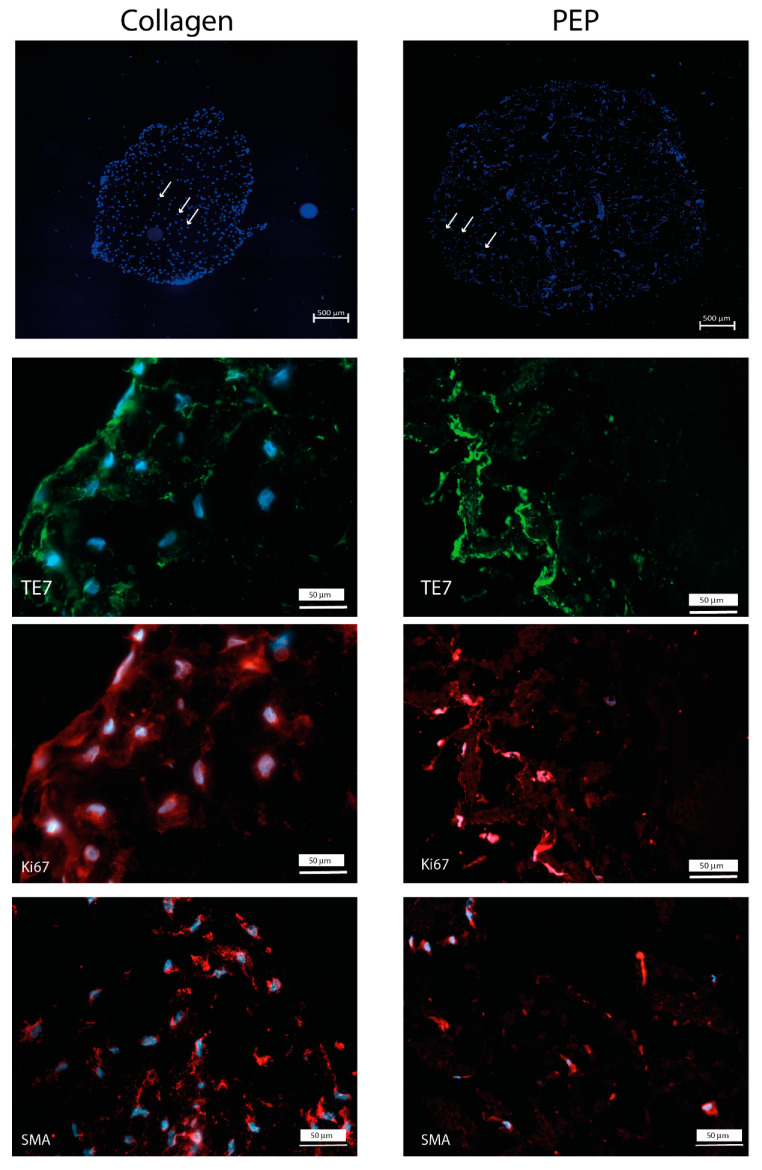
Scaffold with collagen (left panel) and with PEP (right panel) stimulate the growth of fibroblasts. Fibroblasts cells were cultured for one week, and further examined by immunohistochemistry. Expression of fibroblast marker TE7 (light green), proliferation marker KI67 (red), and fibroblast differentiation marker SMA (red). Cell nucleus outlined by Hoechst-staining (blue).

**Figure 4:**
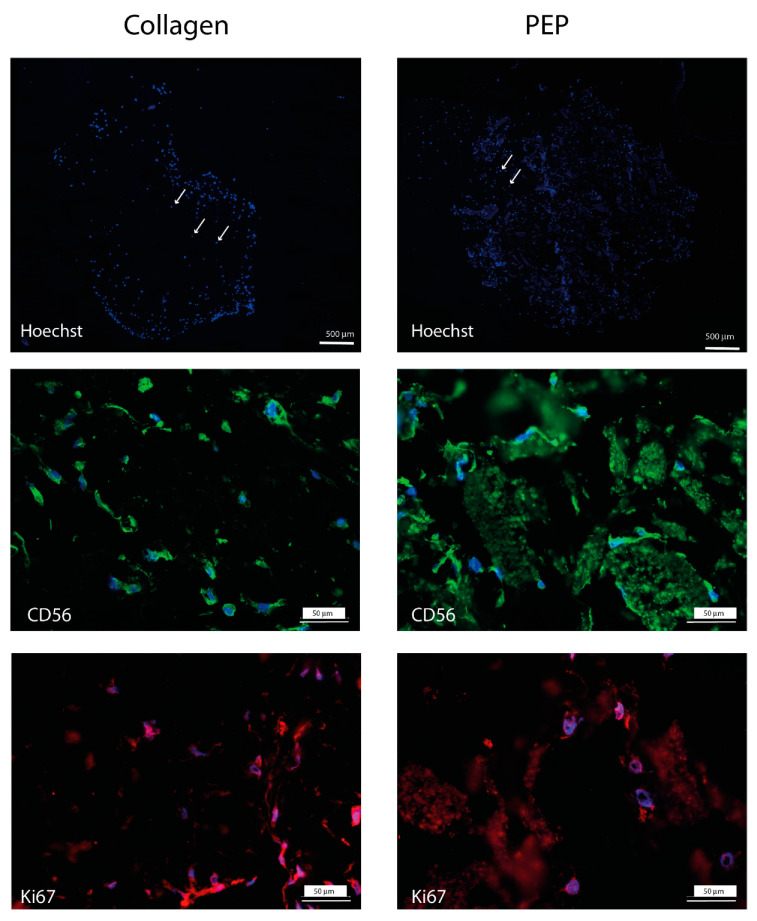
Scaffold with collagen (left panel) and with PEP (right panel) stimulate growth of primary skeletal muscle cells. Primary skeletal muscle cells were cultured for one week, and further examined by immunohistochemistry. Expression of skeletal muscle marker NCAM (light green) localized around nuclear area by Hoechst-staining (blue), together with proliferation marker KI67 (red). Cell nucleus outlined by Hoechst-staining (blue). PEP clusters visualized as diffuse green or a red background.

Cell attachment, migration, and proliferation were examined for fibroblasts and bovine skeletal muscle cell. Both fibroblasts and skeletal muscle cells were able to migrate into and proliferate within the scaffolds and both cell types retained their phenotypic properties visualized by immunostaining (Figure 3 and Figure 4).

**Figure 5:**
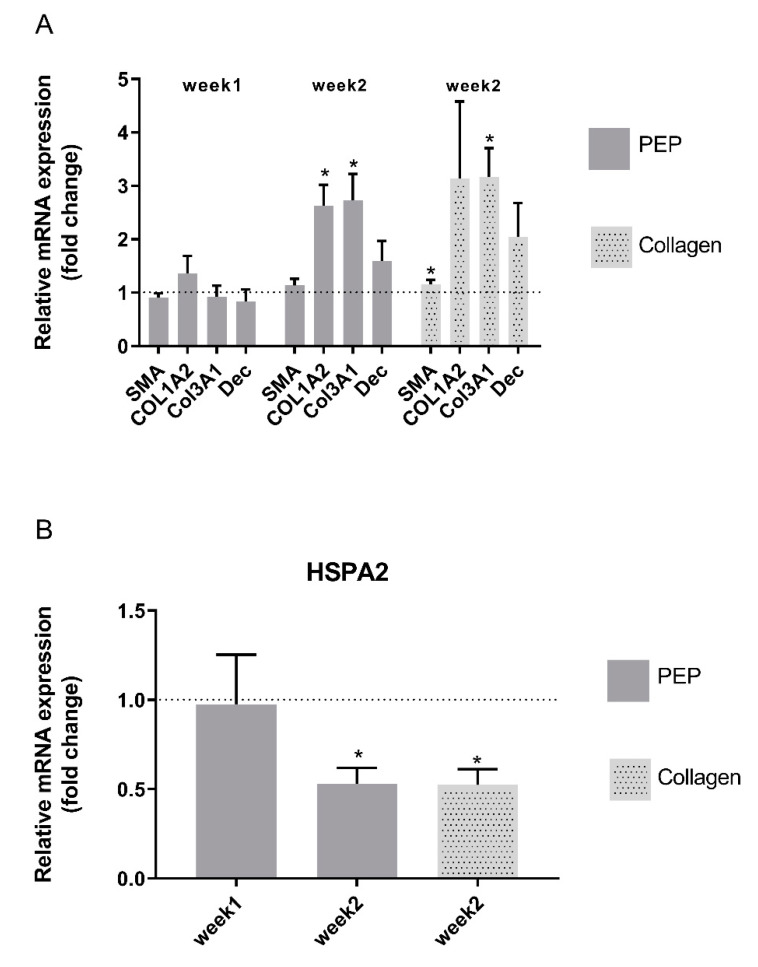
mRNA expression of ECM components and stress markers of fibroblasts cultured in a collagen scaffold with and without PEP. Fibroblasts differentiation and extracellular matrix production (**A**) is increased together with reduced stress marker HSP (**B**) in both scaffolds after long-term culturing for two weeks. Bars shows relative mRNA levels presented as fold change (ΔΔCt) in PEP scaffold/collagen scaffold (week 1). Collagen scaffold (week 1) is set to 1 (represented as dotted line). Data are presented as mean ± stdv from three independent cell experiments seeded out in duplicates * Significant difference compared to fibroblast cultured on a collagen scaffold for one week determined by one-way ANOVA using Dunnett’s multiple comparison test.

Monitoring mRNA expression of fibroblast markers and extracellular matrix components supported viable phenotypes and matrix-producing cells in both types of scaffold (Figure 5). Fibroblast extracellular matrix production was increased after longer term culture for two weeks, however no significant difference in mRNA expression patterns was obtained between the two scaffolds. The stress marker HSP was reduced after longer time culturing, indicating good viable culture conditions for the cells. 

**Figure 6:**
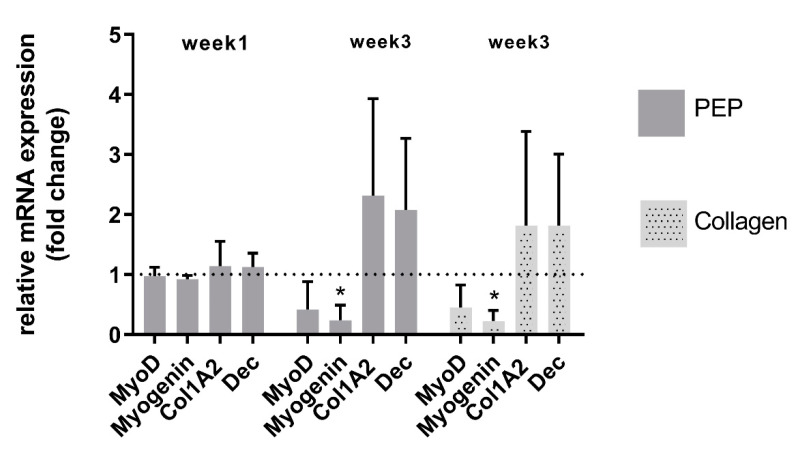
mRNA expression of ECM components and muscle markers of primary skeletal muscle cells cultured in a collagen scaffold with and without PEP. Skeletal ECM production of collagen (Col1A2) and decorin (Dec) was increased after three weeks of differentiation, whereas the differentiation muscle marker myogenin was reduced. Bars shows relative mRNA levels presented as a fold change (ΔΔCt) in a PEP scaffold/collagen scaffold (week 1). Collagen scaffold (week 1) is set to 1 (represented as dotted line). Data are presented as mean ± stdv from three independent cell experiments seeded out in duplicates. * Significant difference compared to primary skeletal muscle cells cultured on collagen scaffold for one week determined by one-way ANOVA using Dunnett’s multiple comparison test.

Monitoring mRNA expression of the skeletal muscle markers myoD and myogenin, as well as extracellular matrix components collagen and decorin supported also viable phenotypes and matrix-producing skeletal muscle cells in both types of scaffolds (Figure 6). An increase in extracellular matrix production of skeletal muscle cells was observed after longer-time culture, and with no significant difference between the two types of scaffold (Figure 6). A decrease in myogenic markers was however observed in both types of scaffold after longer-term culture of these cells.

## 3. Discussion

In this study we show that PEP consisting of highly fibrous proteins such as CREMPs and collagen, and with milled particles of fiber-like structure and cell growth-stimulating properties [7,8,12], could replace the more industrially expensive collagen often used in cryogel scaffolds. The outcome on cell growth, phenotypic marker expression and ECM production of fibroblasts and primary skeletal muscle cells was similar. Both scaffolds supported proliferation detected by Ki67 and expression of the phenotype markers (TE7, SMA, NCAM), differentiation markers (SMA, myoD, myogenin) and extracellular matrix production after short and longer culture time. The expression of primary skeletal muscle cell markers decreased however during the incubation period. The reason for this could be that the seeding density that was used was too low, and we had not determined the optimal number of skeletal muscle cells to be loaded onto the scaffolds. The cell seeding density is a critical parameter for cell proliferation and differentiation in scaffolds [17,18]. We also observed that the distribution of the cells within the construct was not homogenous, with higher cell densities at the borders than in the middle. This could be attributed to the seeding method. The advantage of seeding cells on top of the scaffold, allowing passive infiltration of the scaffold over time, is that the cells are not subjected to a potentially damaging mechanical force and high shear stress that could impact cell viability. However, seeding on top, particularly on thick scaffolds, might influence infiltration rates, resulting in an uneven distribution of cells within the scaffold. One solution could be to use more active seeding technologies, such as centrifugal or external pressure gradients [19,20]. Finally, the supply of oxygen and nutrients is a critical factor in tissue engineering. Our data revealed a porous structure in both scaffolds, allowing the supply of oxygen and good migration potential. We have previously demonstrated in 2D-culture, that the fibroblasts migrated towards and into clusters of PEP. In this study, the fibroblast and skeletal muscle cells were observed both on collagen fibrils and were attached on PEP clusters, supporting our previous suggestion that PEP is not a chemoattractant, and that the cells have to be in proximity or in direct contact with PEP aggregates to become migratory [7]. This visual inspection was also consistent for the primary skeletal muscle cells, and therefore not only fibroblast specific. PEP contains a collagenous matrix in addition to fibronectin, fibrin and vitronectin [9], all markers that are reported to play essential roles in the regenerating healing processes. Our previous mice trial study had revealed a stimulating activity on epithelial and endothelial cells [7,8], suggesting a broad cell-stimulating activity of PEP. The transition from nonmotile to motile cells is determined by soluble growth factors or an extracellular matrix [21]. It has been demonstrated that the collagen matrix initiates dermal fibroblast motility in the absence of any growth factors, but that platelet-derived growth factor (PDGF) does not stimulate migration alone without a collagen matrix [22]. 

ESM consists of a high amount of structural proteins such as cysteine-rich eggshell membrane proteins (CREMP) but also collagen III, IV, V, X, XII [9]. These proteins have previously been identified in PEP [23], and CREMPs and collagen X were the dominating proteins, confirming the structural nature of ESM. CREMPs are cysteine-rich ESM proteins and provide strength to the ESM because of the presence of molecular disulfide linkages in cysteine-rich repetitive domains. Repetitive motifs in a protein contribute to its structural and adhesive functions [24], and CREMP proteins are present in the wall of nematocysts and help to withstand extreme osmotic pressure [25]. The triple helical structure of collagen confers compressive and tensile strength to animal tissues, and analysis of the mechanical properties of ESM is similar to the mechanical behaviour of other tissues, such as tendons that are rich in collagen I [26]. Thus, the increased hardness we observed for the PEP scaffold at 4 h post-swelling can be explained to some extent by the already known mechanical properties of ESM. Moreover, the inclusion of PEP in the scaffold significantly increased the amount of PBS absorption, while the water-holding capacity (i.e., the ability to retain PBS during compression) was higher for the collagen scaffolds, indicating that PEP binds water more loosely compared to collagen. To fully evaluate the potential use of PEP in 3D-constructs, it is thus important to gain more knowledge regarding its ability to retain/release water as well as its mechanical properties. Thus, PEP aggregates in the scaffolds improve important characteristics, including PBS moisture retention, and is a porous structure allowing permeability of nutrition, biocompatibility, cell adhesion and nontoxicity, all of which make it a promising biomaterial for clinical applications. For industrial purposes, ESM material is also easily sterilized by gamma irradiation and is very stable during storage, it is an attractive low-cost biomaterial for production on a large scale.

Collagen is today an ingredient in different wound healing products, and the structural properties of collagen fibers, with effects on cell proliferation and remodeling activities, have been outlined [27]. It is at present unknown if a pure PEP-scaffold would be even more cell-stimulating, and whether other production techniques such as electrospinning or bioprinting would be preferable for this purpose. Interestingly, it has recently been shown that scaffold developed by electrospinning of a mix of poly-caprolactone, silk fibroin, aloe vera, and a soluble eggshell membrane fraction was highly beneficial for keratinocytes [28].

## 4. Materials and Methods

### 4.1. Preparation of Scaffolds

The processed eggshell membrane powder (PEP) used in this study was prepared as previously described [12]. In short, the raw material used for the studies is an industrial eggshell by-product from Nortura, Norway, which is derived from approved food quality hen eggs. The by-product eggshell is processed and the eggshell membrane from this by-product is harvested by the use of a patented process owned by Biovotec (international application: WO 2015/058790). The harvested ESM is washed, purified, dried and milled to small particles under 100 microns and gamma irradiation at 25 kGy before use. The scaffolds were prepared using 0.75 % (*w*/*v*) Bovine collagen type 1 (Collagen Solutions) solubilized in 20 mM acetic acid alone or with 3 % (*w*/*v*) PEP into a homogenous liquid suspension by use of an IKA T18 Turrax Mixer (IKA-Werke GmbH, Breisgau, Germany). The scaffolds were crosslinked by dehydrothermal treatment (DHT)-crosslinking at 105 °C, 50 mbar for 24 h and freeze drying (Virtis Genesis, LabX, Warminster, PA, USA) into rectangular flat sponges. Circular scaffolds 2 mm thick and 5 mm in diameter were prepared from these sponges prepared by using skin biopsy punches and regular scalpels to fit 96-well cull culture dishes. For the cell experiments, the scaffolds were sterilized in ethanol for 10 min, and washed in PBS twice before use. 

### 4.2. Moisture Absorption and Texture Properties 

From each scaffold type, 6 discs of 10 mm diameter were cut and used for the determination of moisture absorption and texture properties. All tests were performed at room temperature. Each scaffold sample was weighed and placed in a small closed container, before 1 mL of PBS was added to assess the absorption capacity of the samples. After 20 min, the excess PBS was removed by pipetting, and the samples were allowed to rest for an additional 15 min before excess PBS was removed again. The scaffolds were then weighed to calculate the amount of PBS that had been absorbed by the scaffolds. A Texture Analyser TA.XT2 (Stable Micro Systems Ltd., Surrey, UK) equipped with a 12.5-mm cylindrical probe was used to determine the texture properties of the swollen scaffolds at 1 and 4 h post-swelling. The probe was pressed down on the scaffolds at a constant speed of 0.5 mm/s, with two-cycle compression to 60% of the original scaffold height. The force (N) necessary to maintain the constant speed of the probe was recorded continuously during compression, resulting in a texture profile curve (force vs. time; Figure 1). The following texture parameters were calculated based on the texture profile curves: Height of the scaffolds; height reduction following compression; hardness as the maximum force during the first compression cycle; cohesiveness defined as the area of work needed during the second compression cycle divided by the area of work during the first compression. Following the TPA measurements, the scaffolds were weighed to calculate their water holding capacity, calculated as the percentage of the PBS retained after the first and second TPA-measurement as compared to the initial PBS content and after the 1 h post-swelling TPA-measurement, respectively.

### 4.3. Scanning Electron Microscopy (SEM)

The prepared scaffolds were mounted on an aluminum stub using a double-sided tape coated with carbon. The sample was then coated with gold/palladium using a Sputter Coater (Polaron SC7640 Sputter Coater, Quorium Technologies, East Sussex, UK) and examined by an Enviromental Scanning Electron Microscope (Zeiss EVO-50-EP, Carl Zeiss, Jena, Germany).

### 4.4. Cell Culturing in Scaffolds 

Human primary dermal fibroblasts (ATCC, Manassas, VA, USA) were cultured in high glucose Dulbecco’s modified Eagle´s medium (DMEM) (CatNo: 1056501810565018) supplemented with 10% fetal bovine serum (FBS), Pencilline/Streptomycine (P/S) (10,000 units/mL) and Amphotericin B (250 μg/mL) (all purchased from Thermo Fisher Scientific, Waltham, MA, USA) in 75 cm2 culture flasks. The cells were maintained at 37 °C in a humidified atmosphere of 5 % CO_2_ and sub-cultivated in culture flasks in a concentration of 5.000 cells/cm2 until 80% confluence before being collected and seeded onto the 3D-scaffolds. Bovine primary skeletal muscle cells (satellite cells) were isolated essentially as described [29,30]. The isolated bovine skeletal muscle cells were further sub-cultivated in proliferation media consisting of DMEM containing 2% FBS, 2% Ultroser, P/S (10,000 units / mL) and Amphotericin B (250 μg/mL) in 75 cm^2^ Entactin-Collagen-Laminin cell attachment matrix (Merck, Kenilworth, NJ, USA) coated culture flasks at a concentration of 5000 cells/cm^2^ until 80% confluence before collection and seeding onto 3D-scaffolds. All cell culture experiments were performed in 2nd or 3rd passage. 

For scaffold experiments, fibroblasts and skeletal muscle cells were seeded on the top of the scaffolds at a concentration of 400,000 cells/scaffold in the proliferation media described above, and cultured for one and two weeks (fibroblasts) or for one and three weeks (skeletal muscle cells) with medium change every second day. Instead of 10% FBS used for fibroblast, a combination of 2% FBS and 2% Ultroser G serum was used in medium for the skeletal muscle cells. The scaffolds were then washed with PBS twice before being embedded in Tissue Tek OCT, frozen in liquid nitrogen and stored at −80 °C for cryosectioning, or stored in RLT-lysis buffer (RNeasy mini kit (Qiagen, Hilden, Germany) for RNA isolation.

### 4.5. Histology and Immunohistochemistry

For characterization of the scaffold structure, sections (5-µm-thick) of paraffine-embedded scaffold samples were cut on a paraffin microtome (Leica RM 2165, Leica Microsystems, Wetzlar, Germany) and mounted on poly-L-lysine-coated glass slides. The histological analysis was carried out on deparaffinized and rehydrated sections: 2 × 5 min in xylene before rehydration in graded baths and then rinsing with dH_2_O. The overall structure was revealed by hematoxylin and eosin (H&E)- staining. The sections were immersed in H&E staining solution at room temperature for 3 min, rinsed in running water, dehydrated in absolute ethanol and mounted in Eukitt. The sections were then examined with a Leica DM6000B microscope (Leica Microsystems, Wetzlar, Germany). 

For the monitoring of cell expression markers by immunohistochemistry, cryo-fixed scaffolds after cell culturing described above were cryo-sectioned and mounted on poly-L-lysine-coated glass slides before being washed three times in PBS and permeabilizing with 0.5% TritonX-100 in PBS for 15 min. After washing in PBS-tween, the sections were blocked using 5% milk in PBS-tween for 1 h before incubation with primary antibodies for 1 h. The primary antibodies used were rabbit anti-α-SMA polyclonal antibody (1:200 dilution, ab 5694, Abcam, Cambridge, UK), mouse-anti TE7 monoclonal antibody (dilution 1:100, CBL271, Millipore, Billerica, MA, USA), or mouse-anti CD56 monoclonal antibody (ab 5.1H11, The developmental Studies Hybridoma Bank, Iowa City, IA, USA), rabbit-anti Ki67 monoclonal antibody (ab16667, Abcam, Cambridge, UK). The cells were washed three times in PBS and subsequently incubated with Alexa 488-conjugated goat-anti-mouse secondary antibody (1:400 dilution; Life Technologies, Carlsbad, CA, USA) or Alexa 546-conjugated goat-anti-rabbit secondary antibody (1:400 dilution; Life Technologies, Carlsbad, CA, USA) for 30 min before mounting using Dako fluorescent mounting medium (Glostrup, Denmark). The cells were examined by Zeiss Axio Observer Z1 microscope (Zeiss, Jena, Germany), and images were processed using Adobe Photoshop CS5.1. Hoechst dye from Molecular Probes (Hoechst 33343, Thermo Fisher, Waltham, MA, USA) was used to counterstain the nuclei. If necessary, an adjustment in brightness and contrast were performed manually across the entire image.

### 4.6. Quantitative Real-Time PCR

Total RNA was isolated and purified using RNeasy mini kit (Qiagen, Hilden, Germany) and with DNAse step (Invitrogen, Carlsbad, CA, USA) according to the manufacturer’s instructions; 200 ng of RNA was subjected to reverse transcriptase (RT)-reaction by Taqman Gold RT-PCR kit (Applied Biosystem, CA, USA). The samples were diluted 4X before application of 10 ng samples (in triplicates) to real-time PCR analysis in an ABI prism 7900HT Sequence detection system (Applied Biosystem), using TaqMan Gene expression assay (Applied Biosystem, San Francisco, CA, USA). Amplification of cDNA by 40 two-step cycles (15 sec at 95 °C for denaturation of DNA, 1 min at 60 °C for primer annealing and extension) was performed, and cycle threshold (Ct) values were obtained graphically (Applied Biosystem, San Francisco, CA, USA, Sequence Detection System, Software version 2.2). Gene expression was normalized to the average value of elongation factor 1 alpha (EF-1α) and ribosomal protein L32 (RPL32) and ΔCt values were calculated. Comparison of gene expression between samples (collagen scaffold (control) and PEP containing scaffold) was derived from subtraction of ΔCt values between the two samples to give a ΔΔCt value, and relative gene expression (fold change) was calculated as 2^-ΔΔ^Ct normalized to control. Applied Biosystem’s primer/probe assays were used in this study: Col1A2(Hs01028939_g1),Col3A1(Hs00943809_m1),Dec(Hs00370384_m1),SMA(Hs00909449_m1),HSPA2(Hs00745797_s1),EEF1A1(Hs00265885_g1),RPL32(Hs00851655_g1),MyoD(Bt03244740_m1),Myogenin(Bt03258928_m1),Col1A1 (Bt03225329_g1), Dec (Hs00370384_m1), EEF1A1 (Bt03223794_g1).

### 4.7. Statistical Analysis

Results from the mRNA analysis were expressed as mean ± standard error mean (SEM) of at least three independent cell seeding experiments on scaffolds performed in technical triplicates. Significant variance by treatments in comparison to the control sample was determined by one-way ANOVA using Dunnett’s multiple comparison test. Differences were considered significant at *p* < 0.05. All statistical analysis was performed in Graph Pad Prism version 7.03 (GraphPadSoftware, La Jolla, CA, USA). Data from the texture profiling were analyzed with factorial ANOVA using the GLM procedure in Minitab (Version 18.1), with the main effects of scaffold type and post-swelling time and their interaction, while Tukey’s pairwise comparison was used to reveal the significant differences between the groups. 

## 5. Conclusions

A low-cost biomaterial with cell-stimulating properties is of interest for tissue engineering. The development of porous structures using natural biopolymers, such as PEP is a promising biomaterial with potential advantages for tissue engineering applications. PEP provides mechanical support as well as a platform for cellular interaction, and hence has the possibility of forming a fully functional tissue in the end. Further optimization regarding long term culture, seeding densities, seeding methods, together with other scaffold production methods would be of interest in further research.

## Figures and Tables

**Table 1:** Physical scaffold properties.

Physical Properties	1 h Post-Swelling		4 h Post-Swelling		SEM	*p* Values		
	Collagen	PEP	Collagen	PEP		Scaffold (S)	Time (T)	S × T
Height swollen, mm	1.50c	2.75a	1.25d	2.28b	0.13	0.000	0.000	0.012
Height reduction following compression, % ^1^	16.4	17.3	-	-	1.34	0.756	-	-
PBS content, mg/mm scaffold	81.2b	123.4a	61.4c	80.0b	4.80	0.000	0.000	0.000
Water-holding capacity, % ^2^	75.0	69.5	84.2	77.2	1.23	0.000	0.000	0.528
Hardness, N	2.19b	2.57b	2.31b	4.44a	0.21	0.000	0.000	0.000

abcd Different letters in the same row indicate significant differences between average values for each scaffold type and time point measurement (*p* < 0.05). ^1^ Percentage reduction in height after TPA-measurements at 1 and 4 h post-swelling. ^2^ Calculated as percentage PBS retained after the 1 h post-swelling TPA-measurement as compared to initial PBS content.

## Abbreviations

PEP	Processed eggshell membrane powder
ESM	Eggshell membrane
ECM	Extracellular matrix
